# Trends in Cancer Incidence and Associated Risk Factors in People Living with and Without HIV in Botswana: A Population-Based Cancer Registry Data Analysis from 1990 to 2021

**DOI:** 10.3390/cancers17142374

**Published:** 2025-07-17

**Authors:** Anikie Mathoma, Gontse Tshisimogo, Benn Sartorius, Saajida Mahomed

**Affiliations:** 1 College of Health Sciences, University of KwaZulu Natal, Durban Private Bag X54001 4000, South Africa; b.sartorius@uq.edu.au (B.S.); mahomeds@ukzn.ac.za (S.M.); 2 Division of Research and Enterprise, University of Botswana, Gaborone Private Bag 0022, Botswana; 3 Ministry of Health, Gaborone Private Bag 0038, Botswana; gtshisimogo@gov.bw; 4 Faculty of Medicine, University of Queensland, Brisbane, QLD 4072, Australia; 5 Centre for Tropical Medicine and Global Health, University of Oxford, Oxford OX1 2JD, UK; 6 Department of Health Metric Sciences, University of Washington, Seattle, WA 98195, USA

**Keywords:** cancer, incidence, HIV, antiretroviral, AIDS-defining cancers, non-AIDS-defining cancers, risk-factors

## Abstract

**Background**: With a high human immunodeficiency virus (HIV) adult prevalence, people living with HIV (PLHIV) in Botswana continue to experience a high burden of comorbid HIV and cancer. We sought to investigate the trends of acquired immunodeficiency syndrome (AIDS) defining cancers (ADCs), non-AIDS defining cancers (NADCs), and associated risk factors in PLHIV compared with those without HIV. **Methods**: We analyzed data from adults aged ≥18 years reported in Botswana National Cancer Registry and National Data Warehouse. The crude, age-standardized incidence rate (ASIR), standardized incidence ratios (SIRs) of cancers and time trends were computed. Risk factors were determined using the Cox-regression model. **Results**: Over a 30-year period, 27,726 cases of cancer were documented. Of these, 13,737 (49.5%) were PLHIV and 3505 (12.6%) were people without HIV and 10,484 (37.8%) had an unknown HIV status. Compared to the HIV-uninfected, the PLHIV had higher and increasing trends in the cancer incidence overall during the study period (from 44.2 to 1047.6 per 100,000; *p*-trend < 0.001) versus (from 1.4 to 27.2 per 100,000; *p*-trend < 0.001). The ASIRs also increased in PLHIV for overall ADCs, NADCs and other sub-types like cervical, lung, breast, and conjunctiva cancers (*p*-trend < 0.001). Further, PLHIV had elevated SIRs for cervical cancer, Kaposi sarcoma in males and some NADCs. The most common risk factors were HIV infection and female sex for ADCs incidence and advanced age and being HIV-uninfected for NADCs incidence. **Conclusions:** Increasing trends of ADCs and NADCs during ART expansion were observed among PLHIV compared to those without HIV highlighting a greater need for targeted effective prevention and screening strategies including the provision of access to timely HIV and cancer treatment.

## Simple Summary

Over the years, the number of cancer cases in Botswana has been rising. Additionally, the country has high HIV and cancer comorbidity. This study sought to determine the cancer trends and associated risk factors during antiretroviral therapy (ART) expansion among people with HIV versus those without HIV. The results showed a high burden of cancer with 27,726 incident cases reported during the study period. Compared with the HIV-uninfected, PLHIV experienced high and increasing trends in the incidence rates of cancer overall, AIDS-defining cancers and some non-AIDS-defining cancers. Furthermore, cancers such as Kaposi Sarcoma and lung among males, cervical, and conjunctiva were found to be in excess in the late post-antiretroviral era. The predictors for cancer were HIV infection, aging, being single and female sex. These findings highlight the need for targeted effective prevention and screening strategies with access to timely cancer treatment combined with early initiation of ART and effective HIV management.

## 1. Introduction

The human immunodeficiency virus (HIV) and acquired immunodeficiency syndrome (AIDS) pandemic remains a global public health burden. It is estimated that 39 million were people living with HIV (PLHIV) by 2022 while 40.4 million people have lost their lives to date [1]. While significant milestones in scaling up combined antiretroviral therapy (cART) over the past two decades have been made, people in low- and middle-income countries (LMICs) are still disproportionately affected by the HIV/AIDS pandemic, with the sub-Saharan Africa (SSA) region accounting for more than two-thirds (25.6 million) of all PLHIV worldwide [1]. Botswana, a middle-income country located in the southern part of SSA, is among the countries most affected by the HIV/AIDS epidemic. According to the 2022 Botswana AIDS Impact Survey V (BAIS V), the country’s estimated adult (15–64 years) HIV prevalence was 20.8% [2].

HIV-related immune suppression combined with coinfection with oncogenic viruses (e.g., human papillomavirus (HPV)) has increased the risk of developing many opportunistic infections and cancers in PLHIV [3,4]. While the link between these cancers and HIV/AIDS is still not well understood, it is suggested that the connection most likely depends on a weakened immune system due to HIV viremia, persistent antigenic stimulation and/or other oncogenic viruses as cofactors [5,6]. In 2020, cancer was identified as the leading cause of death globally, with nearly 10 million deaths and an estimated 19.3 million new cases reported [7]. In Botswana, through the Botswana National Cancer Registry (BNCR) established in 1999, cases of cancer reported over time have escalated. Between 1986 and 2004, only 2000 cancer cases were reported by the registry, and by June 2017, the number had increased to 21,834 registered cases; of these, 7783 (35.6%) constituted AIDS-defining cancers (ADCs), namely, non-Hodgkin lymphoma (NHL), cervical cancer and Kaposi sarcoma (KS) [8].

Evidently, upon ART introduction, the incidence rates of ADCs plummeted [6,9,10,11,12]. However, several studies conducted in Botswana and SSA countries have demonstrated that cancer associated with HIV/AIDS is still prevalent despite the roll out of ART, with PLHIV being at a greater risk of ADCs than the general population is [12,13,14,15,16,17]. In addition, a substantial number of PLHIV in LMICs are not aware of their HIV status, initiate ART late, or are not on ART despite being eligible for treatment [1,18]. Conversely, owing to the aging and longevity of PLHIV on ART, non-AIDS-defining cancers (NADCs), such as lung, anal, hepatocellular and breast cancers, have emerged [19,20,21]. In Botswana, some cases of ADCs, and NADCs remain high [8], with ADCs (cervical cancer and KS) and NADCs (prostate, esophageal and breast cancers) leading in the cause of morbidity in 2022 [22].

While the LMIC region, particularly in SSA, bears the highest burden of comorbid HIV and cancer compared with high-income countries, there is a paucity of data regarding this burden in LMICs [23,24]. In this study, we sought to analyze data from the BNCR to understand the incidence and risk factors associated with the development of ADCs and NADCs during ART expansion comparing PLHIV to those uninfected with HIV. This research is important for Botswana and the region to identify which cancers PLHIV are at increased risk for, and the information can contribute to the development of screening guidelines for common and preventable cancers such as breast cancer and help inform the national cancer control plan (NCCP) currently underway to curb the high morbidity of these cancers.

## 2. Methods and Materials

### 2.1. Study Design, Setting and Population

This was a retrospective analysis of a census of all adult patients aged ≥18 years who were diagnosed with cancer and registered in the Botswana National Cancer Registry (BNCR) from January 1990 to June 2021.

### 2.2. Data Source and Collection

#### 2.2.1. Botswana National Cancer Registry

The BNCR is an International Agency for Research on Cancer (IARC) endorsed population-based and Ministry of Health (MoH) registry established in 1999 to capture national data from all primary health care facilities as well as private and public referral hospitals with oncology centers in the country [25]. The registry collects information based on a monthly cancer notification form completed by the health facilities. The information covers population demographics such as names, age, nationality, marital-status and sex; risk factors namely smoking and alcohol intake; the basis of the diagnosis such as death certificate, clinical, histology and surgery; cancer diagnosis, staging and treatment of cancers in the whole country. The other data captured include HIV status whether positive or negative and with the revised cancer notification form of 2021, a ‘Not Known’ HIV status was added; and finally, ‘present status of the patient’ (dead, alive and not known), date and cause of death.

Notably, over the years, the BNCR data capture has improved with many cancer cases reported in recent years. However, during data extraction for this study, there were substantial amounts of data missing from the registry especially on variables such as HIV status, present status, smoking, alcohol intake and staging, highlighting data entry and quality challenges. This was primarily caused by various health facilities and oncology centers submitting incomplete cancer notification forms with patient data. Furthermore, the problem was compounded by the fact that reporting to the cancer registry is not uniform across all cancer centers [26].

#### 2.2.2. The National Data Warehouse

With most of the patients missing HIV-status data, combined with the need for other variables that are not reported in the BNCR like HIV diagnosis date, CD4 cell count and viral load, permission was sought to access the National Data Warehouse (NDW). The NDW, a MoH database which serves as the repository of all national health data [27], was linked to the BNCR. The repository is fed by a multitude of sources, including the Patient Information Management Information System (PIMS), the Integrated Patient Management System (IPMS) and the Births and Deaths registry amongst others. The PIMS and IPMS databases collect data from patients after initiation of ART and during follow-up visits in HIV care. In the linkage process, the records were compared using the personal national identity (ID) number as the primary identifier to link the cancer data to the rest of the data in the NDW. A match was accepted if there was exact concordance of the national ID with the first and last name and sex. Records with missing IDs or IDs not conforming to the correct criteria, such as those with fewer than 9 digits, were not considered. At the end of the process, around 7189 records mostly belonging to individuals with ‘unknown HIV status’ were not accepted as a true match due to lack of agreement between the national IDs or inconsistency between the ID and other personal identifying information. After identifying the true matches, the names of patients and ID numbers were removed from the final dataset to ensure data privacy.

#### 2.2.3. Antiretroviral Program in Botswana

The Botswana ART program was initiated in 2002 in several urban areas and scaled up nationally by 2004. From inception until early 2008, the criteria for enrolment in the program were PLHIV who had AIDS (WHO stage 3 or 4 HIV illness and/or CD4 200 cells/μL). After this, the eligibility for ART was modified as follows; CD4 count ≤ 350 cells/µL from 2008 to 2012; CD4 count ≤ 500 cells/µL from 2013 to 2015. In 2016, the country adopted and started implementing the WHO ‘Treat All’ policy where all individuals diagnosed with HIV infection are initiated on ART as soon as possible regardless of their disease state or CD4 cell count [28].

### 2.3. Statistical Analysis

The study categorized cancers as ADCs (NHL, KS, and cervical cancer) and NADCs such as lung, prostate, liver, head and neck cancers. To evaluate the temporal trends in cancer incidence, we considered calendar trends in cancer risks reflecting an increasing use of ART at population level by stratifying the study population into pre-ART (1990–2001) and post-ART periods (2002–2007, 2008–2012, 2013–2015, 2016–2021). We examined the frequencies of sociodemographic, behavioral and clinical factors overall and by HIV status and compared variables between PLHIV and those living without HIV using Pearson Chi-square test and Fisher’s Exact tests.

To estimate the population of people living with and without HIV, we used age and sex-specific prevalence from the BAIS conducted by the government of Botswana nationally to measure HIV prevalence in the country. The BAIS were conducted in the years 2001, 2004, 2008, 2013 and 2021. Due to gaps between the years when the BAIS were conducted, linear interpolation was used to estimate HIV prevalence for the years which were missing [29]. Linear interpolation allows for a gradual trend instead of assuming constant prevalence over multiple years. The two populations were derived as:

$$\begin{array}{l} HIV-Population=Total\;Population\times \left(1-HIV\;Prevalence\;Rate\right)\\ {HIV}_{+}\;Population=Total\;Population\times HIV\;Prevalence\;Rate \end{array}$$

For each ART period, we estimated person-years (PY) at risk for HIV-positive and HIV-negative groups by taking the mid-year population in each sex × age stratum (from census counts weighted by HIV prevalence or its complement) and assuming one year of follow-up per person at mid-year. Summing across years in each period yielded total person-years for each HIV status group. The PY for each HIV status group were estimated using age-stratified population data from the 2001, 2011, and 2021 national censuses, along with age-specific HIV prevalence estimates from BAIS. This approach relies on population-level estimates rather than individual HIV seroconversion data, which is a key limitation of this study. The date of HIV diagnosis was missing in more than 80% of PLHIV in this study and as a result, could not be applied in the analysis.

The HIV population at risk for cancer was estimated using data from the 2001, 2011, and 2021 national censuses, and for the years between censuses, populations were estimated by assuming a constant growth rate within each age group [30]. The study assumed that HIV status remains fixed throughout the time at risk of developing cancer suggesting that individuals classified as having HIV infection at cancer diagnosis were assumed to be living with HIV for their entire time at risk [20]. This derives from the limitation of our data where around 80% of the PLHIV in the study did not have the date of HIV diagnosis. Furthermore, almost 40% of the enrollees had unknown HIV status meaning both the BNCR and NDW databases had missing information on these individuals’ HIV status. These are individuals who might have been tested for HIV, but either the data is not reported in the BNCR and/or is captured in the NDW but could not be found because of mismatch of the national ID during the linkage process. Therefore, the HIV-unknown cases were excluded from this analysis due to the data constraints as we were not able to estimate the person-year denominator.

Age-standardized cancer incidence rates (ASIRs) per 100,000 population were calculated using direct standardization based on age groups 18–29, 30–39, 40–49, 50–59, and ≥60 across five time periods. The ASIRs were referenced to the HIV-uninfected population as the study aimed to compare the cancer incidence between the people living with and without HIV. To estimate the expected number of cases among PLHIV, we used age-specific cancer incidence rates from the HIV-uninfected group as reference. Cancer incidence rates were obtained for each age group from the HIV-uninfected population and these rates were expressed as the number of cases per 100,000 person-years.

$$Expected\;cases=\sum_{i}\left({IR}_{i}^{HIV-}\times {PY}_{i}^{HIV+}\right)$$

where

${IR}_{i}^{HIV-}$ is the cancer incidence rates for each age group i from the HIV-uninfected population.

${PY}_{i}^{HIV+}$ is the total person-years contributed by individuals diagnosed with HIV in each age group i.

We assessed time trends in ASIRs, assuming a Poisson regression distribution and a *p*-value of less than 0.05 was deemed statistically significant [31]. To assess the robustness of HIV-uninfected and PLHIV person-years estimates, we performed a sensitivity analysis by adjusting HIV prevalence rates by ±5% and ±10% (Supplementary Table S1). By adjusting HIV prevalence, we demonstrated that the findings were robust with only moderate fluctuations of sensitivity scenarios. For all cancers, sensitivity analysis showed a consistent direction even under ±10% prevalence adjustment, with PLHIV still having significantly elevated cancer risk compared to people without HIV. ADC SIRs remained elevated, suggesting persistent vulnerability among PLHIV despite ART. For NADCs, sensitivity scenarios (±5% and ±10%) depicted minor fluctuations, demonstrating stable NADC estimates across adjusted HIV prevalence levels.

Standardized incidence ratios (SIRs) were determined by dividing the observed number of cases in each time-period by the corresponding expected number of cases, along with their 95% confidence intervals (CI) calculated using Poisson regression.

$$SIR=\frac{Observed\;Cases}{Expected\;Cases}$$

Standardized incidence ratios (SIRs) were also calculated separately for males and females in every ART period. For each cancer type and each ART rollout period, we first counted the number of new cases separately among the individuals with and without HIV. We then calculated the total person-years at risk in each group by combining age- and sex-specific population estimates (from national censuses) with corresponding HIV prevalence (from BAIS and interpolated data). Using the HIV-uninfected group as our reference, we derived the expected number of cases in the HIV group by applying the HIV-uninfected incidence rate to the person-years of PLHIV.

Cox proportional hazard model was used to assess the risk for cancer over time and the influence of variables on the incidence. This regression model was selected because of the (i) ability to test for the interaction between time period and risk factor and (ii) allowance to adjust for potential confounders [32]. Two models were fitted to evaluate the associations between covariates and cancers and estimate hazard ratios (HRs) with 95% confidence limits. The HR gives the ratio of two hazard rates of individuals exposed versus those unexposed to the risk of cancer [32]. To identify risk factors, we initially fit bivariate models with covariates including age, sex, marital status, employment and HIV infection. All the variables with bivariate *p* < 0.25 values [33] were included in the multiple regression model to explore their impact on the HR. All statistical analyses were conducted in STATA, 17.0 SE (Stata Corp., College Station, TX, USA) and R version 4.1.2 (R Core Team, 2021, R Foundation for Statistical Computing, Vienna, Austria).

## 3. Results

### 3.1. Overall

Between 1990 and 2021, 27,726 incidents of cancer were documented. The crude cancer incidence rate was 1175 per 100,000 population. The median age was 52 years (IQR 39–66), and 16,021 (57.8%) were females (Table 1). A total of 13,737 (49.5%) were PLHIV, 3505 (12.6%) were HIV-uninfected people and 10,484 (37.8%) had an unknown HIV status. Of the PLHIV, 8172 (59.4%) were females. Compared with HIV-uninfected individuals, PLHIV were more likely to be younger (median age 42 vs. 61), single (68.3% vs. 43.5%), diagnosed with ADCs (54.3% vs. 20.1%) and unemployed (46.0% vs. 23.4%) *p* value ≤ 0.001. People not infected with HIV were more likely to be diagnosed with NADCs (79.9% vs. 45.7%, *p* < 0.001). The majority of cancers across the two groups were diagnosed at advanced stages III and IV.

### 3.2. Cancer Incidence

When compared with their HIV-uninfected counterparts, the PLHIV experienced high and increasing trends in the overall cancer incidence throughout the entire study period (from 34.7 to 1047.6 per 100,000; *p*-trend < 0.001) versus (from 1.4 to 27.2 per 100,000; *p*-trend < 0.001) (Table 2).

**Figure 1:**
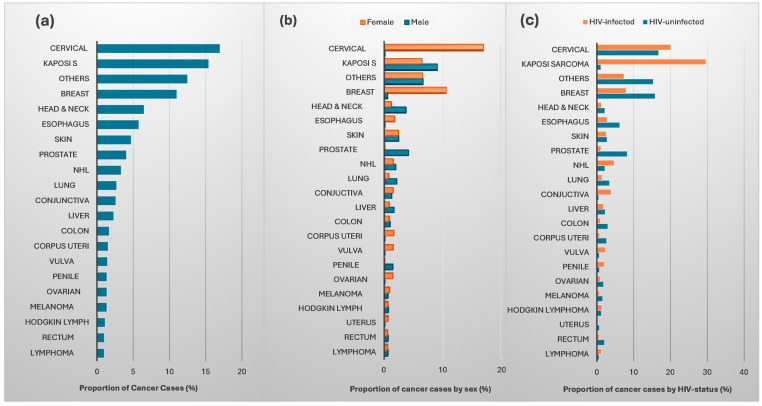
Proportion of individual cancer cases by (**a**) overall, (**b**) sex and (**c**) HIV-status. ‘Others’ include cancers: Autonomic nervous system, biliary, blood, bone marrow, bone cancer, brain, anal, central nervous system, endocrine, eye, female genital tract, renal, gastrointestinal tract, heart, leukemia, lymphoma, lymph nodes, male genital organs, malignant neoplasms, meninges, pancreatic, peritoneum, placenta, rectosigmoid, respiratory, small intestines, spleen, stomach, testes, thymus, thyroid urethra, urinary, uterus, vaginal, other connective tissues and other ill-defined sites.

The most frequently occurring cancers were cervical (17.0%), KS (males: 9.0%; females: 6.4%), breast (males: 0.5%; females: 10.6%), skin including melanoma (males: 2.9%; females: 3.2%), esophageal (males: 4.1%; females: 1.7%), head and neck (males: 3.7%; females: 1.1%) and prostate (4.1%). Among the PLHIV, the most common cancers were KS, cervical, NHL, conjunctiva, penile and vulva (Figure 1).

#### 3.2.1. Incidences of AIDS-Defining Cancers

There were 9904 (35.7%) incident ADC cases from pre- to post-ART, with a crude incidence rate of 420 per 100,000 population. When stratified by HIV status, most of the ADCs were among the PLHIV: 7465 (75.4%). The ADC, with the highest number of cases was cervical cancer: 4703 (17.0%), followed by KS: 4276 (15.4%) and NHL: 925 (3.3%) (Figure 1). After age standardization, an increase in the overall cancer incidence was noted among the PLHIV from pre-ART period 1990–2001 to late post-ART 2016–2021 (from 21.5 to 475.7 per 100,000; *p*-trend < 0.001). Similarly, during the same study period, an upward trend was observed among the PLHIV with cervical cancer (from 6.3 to 301.5 per 100,000; *p*-trend < 0.001). However, during ART expansion from 2002 to 2021 there were significant declines in the incidence of KS (from 603.6 to 127.0 per 100,000; *p*-trend = 0.004) and NHL (from 56.0 to 47.2 per 100,000; *p*-trend < 0.001) (Table 2).

Overall, a decline was observed in the SIRs of all cancers combined among people infected with HIV from 27.40 (95% CI 25.77–29.10) in 1990–2001 to 12.93 (95% CI 12.51–13.36) in 2016–2021). However, during ART expansion, while there was a decrease in the SIRs of all the three ADCs combined from 66.29 (95% CI 63.61–69.93) to 36.84 (95% CI 35.06–36.68), KS from 1449.82 (95% CI 1382.39–1519.69) to 144.29 (95% CI 130.97–158.60) and NHL from 39.23 (95% CI 33.40–45.78) to 31.88 (95% CI 27.13–37.22), during the late ART period 2016–2021, the SIRs showed the incident cases were more than expected especially for KS. On the contrary, from ART inception, the SIR for cervical cancer consistently increased from 13.72 (95% CI 12.41–15.12) in 2002–2007 to 28.57 (95% CI 26.85–30.38) in 2016–2021) (Table 3).

#### 3.2.2. Incidences of Non-AIDS-Defining Cancers

A total of 17,822 (64.3%) NADCs were documented, with a crude incidence rate of 755 per 100,000. Of these, 6272 (35.2%) were individuals infected with HIV. The number of incident cases of breast cancer was the highest: 3055 (17.1%). Other common NADCs (with more than 500 cases) included head and neck (6.5%), esophagus (5.8%), skin (4.7%), prostate (4.1%), lung (2.7%), conjunctiva (2.6%) and hepatocellular (2.3%) (Figure 1). Compared with the HIV-uninfected, the ASIRs for NADCs overall among PLHIV were higher and increased significantly during the post-ART period (from 428.6 to 571.9 per 100,000; *p*-trend < 0.001) versus (from 11.6 to 23.2 per 100,000; *p*-trend < 0.001). Increasing trends were also observed among specific NADCs types including breast (from 3.4 to 122.8 per 100,000; *p*-trend < 0.001), head and neck (from 1.1 to 51.4 per 100,000; *p*-trend < 0.001), and skin, including melanoma (from 1.2 to 70.3 per 100,000; *p*-trend < 0.001) (Table 2).

The overall SIRs of all NADCs combined did not change over time while a decline was noted in the SIRs of breast cancer [from 7.88 (95% CI 6.44–9.55) to 6.84 (95% CI 6.20–7.53)], head and neck cancers [from 20.88 (95% CI 14.28–29.48) to 9.57 (95% CI 8.20–11.11)] and liver [from 11.16 (95% CI 6.24–18.40) to 6.70 (95% CI 4.48–9.62)]. Conversely, the SIRs significantly increased for skin, esophagus, prostate, and lung with the highest SIR observed for conjunctiva [from 97.15 (95% CI 84.27–111.45) to 540.00 (95% CI 412.05–695.11)] (Table 3).

#### 3.2.3. SIRs for Males and Females Living with HIV

As shown in Table 4, during the pre-ART period, men living with HIV had a peak in cancer incidence compared to men without HIV SIR:87.5 (95% CI 79.46–95.9). During the same period women with HIV had a relatively lower SIR:18.2 (95% CI 17.3–19.0). In the early to mid-post-ART rollout (2002–2012) both sexes had a decline in their SIRs although men still had 2–3 times higher SIRs than women. By 2016–2021, the women’s incidence had increased to (SIR: 38.3 CI 35.3–41.5), almost 3 times higher than that of men. For the entire study period, the SIRs of ADCs (especially Kaposi sarcoma) remained more elevated in men than women with sharp increases in SIRs > 1000 noted between 2002 and 2012. A similar pattern was observed where males had elevated SIRs compared to females across all the ART periods for most NADCs subtypes including lung, liver, esophagus, skin including melanoma, head and neck.

### 3.3. Risk Factors Associated with AIDS and Non-AIDS Defining Cancers

The analysis by the Cox model (Table 5), showed that the most common predictive factors associated with the occurrence of ADCs were sex and HIV-infection with females having an increased risk by 35% (adjusted Hazard Ratio (aHR) 1.35, 95% CI 1.27–1.42, *p* < 0.001) while individuals with HIV infection were almost twice more likely to acquire ADCs (aHR 1.73, 95% CI 1.58–1.90, *p* < 0.001). Being single was another risk factor for both ADCs (aHR 1.11, 95% CI 1.03–1.19, *p* = 0.004) and NADCs (aHR 1.16, 95% CI 1.11–1.22, *p*-value < 0.001). The other two important risk factors for NADCs was increasing age with the highest hazard ratio observed among older age ≥ 60 (aHR 523, 95% CI 4.73–5.78), *p* < 0.001. In contrast, being male was significantly associated with a lower risk (aHR 0.89, 95% CI 0.86–0.93) of developing NADC.

## 4. Discussion

This study represents one of the largest and the first to investigate the incidence of cancer using linked population-based data in Botswana for a 31-year period before and after the introduction of ART. The overall cancer incidence of both the people living with and without HIV increased consistently over the entire study period, highlighting high cancer burden in the country. As expected, the age-standardized incidences during ART expansion among the PLHIV were greater than those among the HIV-uninfected reflecting the double burden of infectious diseases and non-communicable diseases that the country is facing. There are possible explanations for this increasing trend. First, the country’s HIV prevalence remains high [2] and continues to fuel cancer morbidity in PLHIV. Further, with the expansion of the ART program over the years, many people initiated on ART are in care leading to better and increased detection of cancer cases. This reasoning can also be attributed to the improvement of the country’s health care system over time through targeted initiatives such as clinician training and laboratory diagnostic capacity that have enhanced the use of histopathology services leading to better cancer diagnosis [34]. Another factor that could have contributed to the surge is that over time, coverage and reporting of cases in the cancer registry has improved with more cases reported in the post-ART era compared to the early years of the registry’s establishment. Additionally, in support of this upward trend among PLHIV, a study conducted during the early ART period (2003–2008) in Botswana reported no decline in the number of cancer cases among an enlarging and aging population of PLHIV [12]. Other studies performed in the United States of America have also demonstrated that, compared with the general population, and despite the effectiveness of ART, PLHIV continue to have an increased risk of developing cancers [3,4].

Of all the ADCs reported, three-quarters were among the PLHIV. The high concentration of ADCs in this group is not surprising as it aligns with previous African studies performed in Botswana, Mozambique and Uganda which noted high ADC incidence among PLHIV despite ART expansion [12,30,35]. Furthermore, the study revealed that while the ASIRs and SIRs for the three ADCs combined, and specifically NHL and KS among PLHIV declined during ART scale up, these were still higher than among people who are not infected with HIV. This finding proves that despite being effective, combined ART does not restore the immune system function fully. This was more evident in the late post-ART period (2016–2021) where ADCs were still in excess, especially KS. To further substantiate this finding, some studies have demonstrated that despite the dramatic decline after ART introduction, KS and NHL incidence remained elevated in PLHIV [12,35,36] emphasizing the need for earlier ART initiation.

In contrast, cervical cancer, the leading ADC with the highest number of incident cases, showed a significant increasing trend of both ASIRs and SIRs consistently from pre-to post-ART. In 2022, when ranked by the number of cases, cervical cancer was the lead cancer among women in Botswana [22]. Additionally, the high incident cases of cervical cancer among women in Botswana during ART scale up can be explained by the existence of the national cervical cancer program and guidelines that have improved screening and detection of the new cases. This result is in keeping with studies performed in Botswana, Kenya, and Uganda (African countries SSA), where cervical cancer was the number one cause of morbidity in females [12,16,17]. Moreover, in Botswana and elsewhere in SSA, cervical cancer was identified as the most common cancer affecting women [37], and among women living with HIV, the risk of developing this cancer was almost five times greater than that in women without HIV [16]. In a narrative review that assessed cancer trends, of the seven studies that reported cervical cancer as the most common ADC, five were performed in SSA [38].

Most of the cancers in our study were NADCs, and one-third of these were found in the PLHIV. The leading NADC was breast cancer with more >90% of the incident cases observed among women. Recent data has shown that breast cancer is the second most common cancer after cervical cancer among women in the country [22]. Compared with the HIV-uninfected, PLHIV experienced an upward trend of ASIRs for all NADCs combined and specific types including breast, head and neck, skin and melanoma, esophagus and lung cancers from the pre- to post-ART periods. The increasing trends in NADCs such as conjunctiva, lung, and head and neck cancers are consistent with other SSA studies [30,39]. Moreover, the sharp rise in conjunctival cancer among PLHIV has been reported and attributed to immune suppression and chronic UV exposure [12,30]. Similarly, the rising incidence of lung cancer among PLHIV has been demonstrated [40,41] with smoking, chronic inflammation, and immune dysregulation identified as the most likely contributors [36]. In contrast, for individuals without HIV, only prostate cancer showed an increasing trend in incidence among people aged ≥60 years. Prostate cancer is common in Botswana and was the number one cancer among males in Botswana regardless of HIV status in 2022 [22].

Additionally, although the SIRs for breast, liver, head and neck cancers declined over the study period, there was a significant increase in the SIRs for skin, esophagus, prostate, and lung with the highest SIR observed for conjunctiva, especially in the ART periods 2013–2015 and 2016–2021. The drastic increase in most of the NADCs during these two ART periods can be attributed to two factors: (i) expanded access to ART in Botswana occurred during these ART periods, especially in 2016, when the country adopted the WHO ‘Treat all with ART regardless of CD4 cell count’ policy; and (ii) the country had exceeded the United Nations AIDS (UNAIDS) 95–95–95 set targets, as evidenced by 95.1% of adult PLHIV being aware of their status, 98.0% receiving ART and 97.9% with viral load suppression [2] suggesting that with more PLHIV receiving ART and living longer because of improved immunogenicity, the emergence of NADCs had become inevitable. This is also confirmed by data from several studies showing that cancers such as lung, hepatocellular and oropharyngeal cancers seemed to occur more frequently in PLHIV with prolonged exposure to ART, who were virologically suppressed and living longer [10,19,20,21,42,43,44].

Compared with their male counterparts, women with HIV had excess cancer overall in the late post-ART period possibly owing to a high incidence of cervical and breast cancers observed in this study. These findings align with the recent data showing that cervical cancer followed by breast cancer were leading in the number of new cases and related deaths among women in Botswana regardless of the HIV status [45]. The high incidence of Kaposi sarcoma among males in this study was also not surprising because this cancer had been identified as the most common cancer in men living with HIV especially in SSA [46,47]. This is also supported by 2022 data where Kaposi sarcoma remained among the top three cancers responsible for high incidence and mortality in males in Botswana [45].

Our analysis of risk factors showed strong predictors for ADCs were HIV infection and female sex. People infected with HIV are at higher risk of ADCs because infection with HIV weakens the immune system and in turn reduces the body’s ability to fight viral infections that may lead to KS, NHL and cervical cancer [4,10,19]. There is ample evidence that when compared with people who do not have HIV, PLHIV are substantially at a higher risk of developing ADCs [10,48,49]. Another factor, female sex as a risk for ADCs has two possible explanations: Two-thirds of the women in our study were living with HIV; and cervical cancer, the leading ADC amongst women had the highest number of incident cases in the study. This finding agrees with several studies performed in SSA that found females with HIV were susceptible to ADCs, particularly cervical cancer [24,49,50,51] and multiple factors such as increased vulnerability to HPV infection, risk of co-infection with HIV and HPV and HIV-induced immunosuppression have been identified as the root causes [52]. Even in USA, where incidence is relatively low, when contrasted with women without HIV, the incidence of cervical cancer in women living with HIV was 66% higher [53].

An important risk factor associated with both ADCs and NADCs occurrence was singlehood status. Some studies have demonstrated that compared with their married counterparts, single people are more likely to seek care late and be diagnosed with advanced cancer [54,55], possibly owing to their low socioeconomic status and lack of spousal support, subsequently leading to poor access to health care [56]. The other predictor for NADCs was advancing age with people aged ≥60 years bearing the highest risk. With aging, cancer risk naturally increases. This is why as the aging population with HIV survives longer, HIV/AIDS has become a chronic condition predisposing them to malignancies such as lung and breast cancers [9,12,57]. This was also confirmed by a USA-based study conducted over a 15-year period that concluded increases in the rates of NADCs were driven mainly by the growth and aging of PLHIV [20]. Finally, people not infected with HIV had an increased risk of NADCs and this is in keeping with data that NADCs remain prevalent in this group with evidence from some studies showing high incidence of NADCs among the people without HIV versus those living with HIV [24,58].

### 4.1. Strength

The use of population-based registries in this study offered a unique opportunity to characterize cancer incidence at the population level. Botswana cancer registry considers all types of cancers using multiple data sources from various health facilities.

### 4.2. Limitations

The limitations are primarily related to the retrospective design of our study. First, nearly 40% of this cohort was missing HIV data, which may have resulted in an underestimation of cancer cases in both people living with or without HIV. This gap has the potential to affect the estimation of epidemiological parameters or fail to provide more realistic estimates of incidence in populations and thus poses a challenge for decision making on resource allocation. As a result of this limitation, we could not compare the HIV-unknown and HIV-uninfected because of the difficulty in estimating the denominator population for the HIV-unknown category. Unlike HIV prevalence which provides an estimate of known HIV status distribution in the population, there is no reliable way to determine the actual number of people classified as HIV-unknown. The second limitation is that the data for key variables such as smoking, alcohol use, cancer staging, and date of HIV diagnosis were lacking for approximately 60% to 80% of the individuals. Consequently, most of these variables were not included in some analyses, e.g., covariates such as smoking and alcohol, which are important behavioral risk factors for cancer incidence and mortality, were not included in our regression analysis. Of concern was, smoking, which we omitted in the analysis, was crucial to determine any association with the increasing trends of lung cancer in our study. Finally, the two registries, BNCR and NDW, were not linked, and during the record-linkage process, approximately over 7000 records with missing or incorrect personal information, such as identification numbers, were dropped from this process. Most of these records were for individuals whose HIV status was unknown, and as highlighted earlier, this group was dropped from some analyses. The development of a single national registry is therefore recommended to enable an effective reporting system with all the relevant routine data to inform the epidemiology of different cancers and vital status of both the PLHIV and the general populations.

## 5. Conclusions

Overall, our study found an increasing trend of cancer incidence during ART expansion. The high incident rates of cancer among the PLHIV combined with more than expected number of all three ADCs and some NADCs during the late post-ART era suggest three important things: the role that immunosuppression plays in predisposing those with HIV infection to cancer; the effectiveness of ART in increasing survival and longevity, subsequently fueling incidence of NADCs; and finally, a rise in the number of cancer cases diagnosed with cancer in late ART, most likely owing to an increase in the number of people that were diagnosed with HIV and started on ART same day in the late ART period because of the implementation of the WHO ‘test and treat all policy’. These findings present opportunities to reduce the incidence of cancer through targeted and effective screening strategies and provide access to timely cancer treatment, especially now, as Botswana develops a national cancer control plan. For example, the need to develop national screening guidelines for common cancers like breast and strengthen the HPV vaccination and cervical cancer screening programs cannot be overemphasized. Focused public efforts must continue to emphasize and strengthen strategies for reducing HIV transmission like promotion of condom use, offering Pre-exposure prophylaxis (PrEP) to individuals at high risk, early initiation of ART and effective HIV management coupled with good adherence so that PLHIV are given the same chances of survival as those reported in the general population. Finally, risk factors linked to cancer incidence, including HIV infection, aging, singlehood and female sex, must be addressed early in HIV care by specialists and oncologists to ensure integrated timely and effective HIV and cancer treatment and subsequent improved prognosis after ADC and NADC detection.

## Figures and Tables

**Table 1:** Demographic, behavioral, and clinical characteristics of participants by HIV status.

	All Patients N = 27,726	Patients Without HIV N = 3505	Patients with HIV N = 13,737	*p* Value *
	n (%)			
**Cancer diagnosis age, *median (IQR)***	52 (39–66)	61 (50–72)	42 (34–52)	<0.001 †
**Cancer diagnosis age** ***(years)***				
18–29	2277 (8.2%)	207 (5.9%)	1578 (11.5%)	<0.001
30–39	5063 (18.3%)	244 (7.0%)	4092 (29.8%)	
40–49	5408 (19.5%)	419 (12.0%)	3841 (28.0%)	
50–59	5058 (18.2%)	729 (20.8%)	2394 (17.4%)	
≥60	9920 (35.8%)	1906 (54.4%)	1832 (13.3%)	
**Sex**				
Female	16,021 (57.8%)	2075 (59.2%)	8172 (59.5%)	0.757
Male	11,705 (42.2%)	1430 (40.8%)	5565 (40.5%)	
**Marital Status**				
Married	8341 (30.1%)	1572 (44.8%)	2828 (20.6%)	<0.001
Single	14,815 (53.4%)	1523 (43.5%)	9388 (68.3%)	
Previously married	288 (1.0%)	66 (1.9%)	104 (0.8%)	
Other (separated and cohabitation)	1367 (4.9%)	283 (8.1%)	352 (2.6%)	
Missing	2915 (10.5%)	61 (1.7%)	1065 (7.8%)	
**Employed**				
Yes	5186 (18.7%)	677 (19.3%)	2665 (19.4%)	<0.001
No	10,270 (37.0%)	821 (23.4%)	6313 (46.0%)	
Other	5432 (19.6%)	984 (28.1%)	1013 (7.4%)	
Missing	6838 (24.7%)	1023 (29.2%)	3746 (27.3%)	
**Alcohol intake**				
Current	814 (2.9%)	123 (3.5%)	359 (2.6%)	<0.001
Past	1639 (5.9%)	225 (6.4%)	850 (6.2%)	
Never	3583 (12.9%)	754 (21.5%)	1685 (12.3%)	
Missing	21,690 (78.2%)	2403 (68.6%)	10,843 (78.9%)	
**Smoking**				
Current	859 (3.1%)	124 (3.5%)	359 (2.6%)	0.059
Past	1042 (3.8%)	197 (5.6%)	497 (3.6%)	
Never	4169 (11.4%)	789 (22.5%)	2049 (14.9%)	
Missing	21,656 (81.7%)	2395 (68.3%)	10,832 (78.9%)	
**Cancer category**				
ADCs	9904 (35.7%)	704 (20.1%)	7465 (54.3%)	<0.001
NADCs	17,822 (64.3%)	2801 (79.9%)	6272 (45.7%)	
**ART period**				
<2002	2510 (9.1%)	96 (2.7%)	1057 (7.7%)	<0.001
2002–2007	7937 (28.6%)	607 (17.3%)	3531 (25.7%)	
2008–2012	7124 (25.7%)	943 (26.9%)	3559 (25.9%)	
2013–2015	3798 (13.7%)	817 (23.3%)	2042 (14.9%)	
2016–2021	6357 (22.9%)	1042 (29.7%)	3548 (25.8%)	
**Cancer staging**				
Early staging (I and II)	1710 (6.2%)	372 (10.6%)	964 (7.0%)	0.004
Late staging (III and IV)	3076 (11.1%)	708 (20.2%)	1423 (10.4%)	
Missing	22,940 (82.7%)	2425 (69.2%)	11,350 (82.6%)	
**Outcome status**				
Alive	17,574 (63.4%)	2266 (64.7%)	9004 (65.5%)	0.412
Dead	9609 (34.7%)	1183 (33.8%)	4548 (33.1%)	
Missing	543 (2.0%)	56 (1.6%)	185 (1.3%)	
**Initial CD4 count,** ***median (IQR)***			89 (167–262)	
**Initial CD4 count** ***(cells/mm^3^)***				
<200			1426 (10.4%)	
200–349			587 (4.3%)	
350–500			161 (1.2%)	
>500			150 (1.1%)	
Missing			11,413 (83.2%)	
**Recent CD4 count,** ***median (IQR)***			459 (277–672)	
**Recent CD4 count** ***(cells/mm^3^)***				
<200			802 (5.8%)	
200–349			1111 (8.1%)	
350–500			1126 (8.2%)	
>500			2437 (17.7%)	
Missing			8261 (60.1%)	
**Initial viral load** ***(copies/mL)***				
<400 (undetectable)			2288 (16.6%)	
≥400			2932 (21.3%)	
Missing			8517 (62.0%)	
**Recent viral load** ***(copies/mL)***				
<400 (undetectable)			4534 (33.0%)	
≥400			665 (4.8%)	
Missing			137 (66.5%)	

HIV: Human immunodeficiency virus; IQR: Interquartile range; ART: Antiretroviral therapy; ADCs: AIDS-defining cancers; NADCs: Non-AIDS-defining cancers. * Pearson’s or Fisher’s exact test (all *p* values except for the medians). † Ranksum test of sorts.

**Table 2:** Age-standardized incidence per 100,000 of common cancers in people living with and without HIV from 1990 to 2021 in Botswana.

Cancer	Living with HIV							Living Without HIV						
	Age Group	1990–2001	2002–2007	2008–2012	2013–2015	2016–2021	*p*-Value †	Age Group	1990–2001	2002–2007	2008–2012	2013–2015	2016–2021	*p*-Value †
ALL	18–29	31.61	516.44	88.78	108.65	114.52	0.010	18–29	0.59	3.12	2.93	4.87	2.39	0.244
	30–39	36.26	1304.52	199.21	358.78	380.48	0.309	30–39	1.59	4.82	5.31	7.63	6.20	0.220
	40–49	34.92	1947.31	288.00	714.04	1146.75	<0.001	40–49	1.72	15.44	19.19	23.20	10.75	0.021
	50–59	43.38	3024.62	449.37	1316.85	2179.68	<0.001	50–59	3.00	32.75	45.81	53.06	25.69	<0.001
	≥60	44.19	3294.69	713.66	2760.66	4825.81	<0.001	≥60	1.46	51.29	109.96	139.29	76.39	<0.001
	**Overall**	**34.74**	**1227.05**	**233.59**	**539.63**	**1047.59**	**<0.001**	**Overall**	**1.38**	**15.27**	**23.06**	**31.10**	**27.22**	**<0.001**
ADCs	18–29	21.31	383.81	61.72	75.39	75.61	0.006	18–29	0.08	0.20	0.18	0.70	0.10	0.718
	30–39	23.35	974.33	128.32	204.92	228.40	0.058	30–39	1.06	1.18	1.77	1.33	0.95	0.962
	40–49	19.53	1241.70	164.31	378.02	585.24	<0.001	40–49	0.86	4.60	4.63	7.37	2.19	0.263
	50–59	19.61	1488.11	193.40	445.41	820.52	<0.001	50–59	2.35	7.97	10.78	8.58	4.12	0.364
	≥60	17.67	1086.03	213.94	704.17	1328.75	<0.001	≥60	1.66	12.82	24.48	26.97	10.77	0.002
	**Overall**	**21.49**	**799.83**	**128.18**	**250.52**	**475.67**	**<0.001**	**Overall**	**0.67**	**3.70**	**5.18**	**6.09**	**4.00**	0.051
CERVICAL	18–29	7.37	32.33	7.08	12.20	15.57	0.904	18–29	0.04	0.07	0.00	0.20	0.00	0.943
	30–39	5.12	148.33	38.18	86.82	121.78	<0.001	30–39	0.33	1.06	1.33	0.83	0.73	0.954
	40–49	6.90	252.00	63.55	207.74	404.47	<0.001	40–49	0.26	4.01	4.28	6.00	1.87	0.281
	50–59	5.94	423.45	93.15	316.30	566.55	<0.001	50–59	1.17	6.64	9.21	8.58	3.57	0.368
	≥60	3.31	463.70	95.79	448.11	951.94	<0.001	≥60	0.62	11.29	21.78	22.23	8.81	0.005
	**Overall**	**6.34**	**140.20**	**42.93**	**127.90**	**301.46**	**<0.001**	**Overall**	**0.34**	**15.27**	**4.48**	**4.99**	**3.27**	**<0.001**
KS	18–29	13.15	327.44	47.55	53.22	48.92	<0.001	18–29	0.04	0.00	0.06	0.10	0.00	0.971
	30–39	17.64	761.99	78.52	94.48	88.11	<0.001	30–39	0.26	0.12	0.33	0.50	0.07	0.996
	40–49	12.43	882.02	79.98	137.36	126.91	<0.001	40–49	0.17	0.00	0.00	0.27	0.11	0.951
	50–59	13.07	955.78	76.08	87.14	159.08	<0.001	50–59	0.00	0.22	0.22	0.00	0.27	0.750
	≥60	14.36	573.52	103.78	192.05	218.15	<0.001	≥60	0.21	0.57	0.41	1.19	0.98	0.425
	**Overall**	**14.56**	**603.61**	**71.61**	**97.25**	**126.96**	**0.004**	**Overall**	**0.10**	**0.13**	**0.17**	**0.34**	**0.31**	0.105
NHL	18–29	0.79	24.04	7.08	9.98	11.12	0.228	18–29	0.00	0.14	0.12	0.40	0.10	0.691
	30–39	0.59	64.01	11.62	23.62	18.52	0.428	30–39	0.13	0.00	0.11	0.00	0.15	0.984
	40–49	0.20	107.67	20.77	32.92	53.87	0.007	40–49	0.17	0.59	0.34	1.09	0.22	0.742
	50–59	0.59	108.89	24.18	41.96	94.89	<0.001	50–59	0.00	1.11	1.35	0.00	0.22	0.979
	≥60	0.00	48.81	14.37	64.02	158.66	<0.001	≥60	0.21	0.96	2.28	3.56	0.98	0.254
	**Overall**	**0.59**	**56.01**	**13.65**	**25.37**	**47.24**	**<0.001**	**Overall**	**0.06**	**0.40**	**0.54**	**0.76**	**0.42**	0.633
NADCs	18–29	10.30	135.95	27.06	33.26	38.91	0.603	18–29	0.51	2.91	2.75	4.18	2.29	0.268
	30–39	12.91	330.19	70.88	153.85	152.08	<0.001	30–39	2.12	3.65	3.54	6.30	5.25	0.172
	40–49	15.39	705.61	123.70	336.02	561.51	<0.001	40–49	2.57	10.84	14.57	15.83	8.56	0.043
	50–59	23.77	1536.51	255.97	871.45	1359.16	<0.001	50–59	3.65	24.79	35.03	44.48	21.57	<0.001
	≥60	26.51	2208.66	499.72	2056.49	3497.06	<0.001	≥60	1.25	38.46	85.48	112.32	65.62	<0.001
	**Overall**	**13.24**	**428.62**	**105.41**	**289.11**	**571.92**	**<0.001**	**Overall**	**1.34**	**11.58**	**17.88**	**25.01**	**23.22**	**<0.001**
BREAST	18–29	2.85	15.75	2.78	3.33	2.22	0.184	18–29	0.20	0.20	0.24	0.20	0.25	0.943
	30–39	3.65	37.59	11.29	23.62	32.55	0.001	30–39	0.93	1.53	1.44	1.82	1.60	0.642
	40–49	3.95	132.88	20.15	70.38	144.26	<0.001	40–49	0.00	4.16	6.34	4.37	3.29	0.148
	50–59	4.75	217.77	35.55	154.92	265.13	<0.001	50–59	0.78	5.09	7.63	13.34	5.22	0.021
	≥60	3.31	231.85	51.09	248.06	680.90	<0.001	≥60	0.00	4.02	12.86	14.82	10.41	<0.001
	**Overall**	**3.42**	**58.80**	**14.83**	**47.83**	**122.83**	**<0.001**	**Overall**	**0.31**	**2.21**	**3.67**	**4.61**	**4.70**	0.781
HEAD and NECK	18–29	0.48	8.29	1.52	0.00	4.45	0.809	18–29	0.00	0.41	0.48	0.40	0.30	0.674
	30–39	0.89	20.32	3.49	4.47	8.42	0.665	30–39	0.13	0.12	0.33	1.00	0.36	0.516
	40–49	2.17	54.98	11.47	23.84	40.17	0.001	40–49	0.00	1.93	1.71	1.36	0.33	0.76
	50–59	1.19	187.53	37.68	103.28	161.87	<0.001	50–59	0.00	4.43	4.04	7.31	2.20	0.141
	≥60	4.42	256.25	71.84	320.08	350.37	<0.001	≥60	0.21	4.78	7.68	17.49	6.37	0.003
	**Overall**	**1.05**	**36.88**	**10.63**	**26.43**	**51.38**	**<0.001**	**Overall**	**0.04**	**1.64**	**1.86**	**3.69**	**2.14**	**0.035**
SKIN *	18–29	1.03	13.26	3.29	3.33	10.01	0.202	18–29	0.04	0.27	0.12	0.29	0.20	0.819
	30–39	1.18	32.51	6.97	19.79	21.32	0.010	30–39	0.00	0.12	0.22	0.33	0.29	0.575
	40–49	1.18	64.15	11.16	40.87	62.09	<0.001	40–49	0.34	0.30	0.69	1.09	0.33	0.725
	50–59	1.19	96.79	24.89	77.46	156.29	<0.001	50–59	0.26	2.43	2.25	2.22	1.51	0.457
	≥60	2.21	195.24	46.30	240.06	442.92	<0.001	≥60	0.00	2.87	7.88	8.89	3.80	0.028
	**Overall**	**1.15**	**37.57**	**10.17**	**32.77**	**70.27**	**<0.001**	**Overall**	**0.08**	**0.83**	**1.37**	**1.71**	**1.38**	0.628
ESOPHAGUS	18–29	0.24	0.83	0.25	0.00	0.00	0.525	18–29	0.00	0.00	0.06	0.00	0.00	0.964
	30–39	0.49	4.06	1.16	1.92	0.56	0.834	30–39	0.13	0.00	0.00	0.17	0.00	0.854
	40–49	0.79	27.49	6.20	15.89	18.26	0.017	40–49	0.00	0.45	0.34	0.27	0.22	0.789
	50–59	1.19	139.13	27.73	112.97	97.68	<0.001	50–59	0.00	1.11	3.82	3.50	0.96	0.226
	≥60	2.21	231.85	47.90	336.08	396.64	<0.001	≥60	0.42	4.02	10.17	16.30	4.90	0.007
	**Overall**	**0.53**	**20.53**	**6.37**	**24.84**	**34.25**	**<0.001**	**Overall**	**0.06**	**0.73**	**1.69**	**2.59**	**1.28**	0.273
PROSTATE	18–29	0.00	0.00	0.00	0.00	0.00	1.000	18–29	0.00	0.00	0.00	0.00	0.00	1.000
	30–39	0.10	0.00	0.00	0.00	0.00	1.000	30–39	0.26	0.00	0.00	0.00	0.00	1.000
	40–49	0.59	2.29	0.00	0.00	4.57	0.199	40–49	0.69	0.00	0.34	0.00	0.22	0.504
	50–59	3.57	36.30	2.13	6.46	44.65	<0.001	50–59	1.04	0.66	1.80	2.54	1.10	0.593
	≥60	2.21	134.23	36,72	176.04	323.92	<0.001	≥60	0.00	4.40	11.41	12.45	15.67	**<0.001**
	**Overall**	**0.39**	**6.26**	**1.71**	**6.34**	**20.67**	0.108	**Overall**	**0.21**	**0.65**	**1.59**	**1.90**	**3.61**	0.137
CONJUCTIVA	18–29	0.16	24.87	3.04	5.54	0.00	0.153	18–29	0.00	0.00	0.06	0.00	0.00	0.964
	30–39	0.39	98.55	13.61	20.43	6.73	0.035	30–39	0.00	0.12	0.00	0.00	0.00	0.867
	40–49	0.00	107.67	16.43	23.84	21.00	0.531	40–49	0.00	0.30	0.34	0.00	0.00	0.916
	50–59	0.00	139.13	14.93	32.28	44.65	0.458	50–59	0.00	0.44	0.00	0.32	0.00	0.997
	≥60	0.00	85.42	25.54	32.01	59.50	<0.001	≥60	0.00	0.38	1.24	0.00	0.12	0.883
	**Overall**	**0.20**	**70.97**	**12.08**	**19.03**	**17.72**	0.877	**Overall**	**0.00**	**0.18**	**0.22**	**0.04**	**0.03**	0.070
LUNG	18–29	0.24	0.83	0.00	1.11	1.11	0.464	18–29	0.08	0.07	0.12	0.00	0.00	0.793
	30–39	0.39	0.00	0.66	1.92	1.68	0.200	30–39	0.00	0.00	0.11	0.00	0.07	0.803
	40–49	0.39	16.04	1.55	6.81	10.04	0.134	40–49	0.17	0.30	0.69	0.55	0.11	0.874
	50–59	1.19	108.89	12.80	48.41	61.40	<0.001	50–59	0.00	0.66	3.82	0.64	0.96	0.416
	≥60	2.21	146.43	39.91	56.01	132.21	<0.001	≥60	0.00	2.49	4.77	5.04	2.69	0.101
	**Overall**	**0.43**	**13.22**	**3.41**	**8.46**	**16.83**	**0.002**	**Overall**	**0.06**	**0.48**	**1.15**	**0.80**	**0.81**	0.417
LIVER *	18–29	0.32	1.66	0.25	0.00	0.00	0.400	18–29	0.00	0.07	0.06	0.20	0.00	0.844
	30–39	0.30	9.14	1.99	5.11	2.24	0,705	30–39	0.13	0.00	0.00	0.00	0.07	0.792
	40–49	0.99	48.11	5.89	14.76	8.22	0.638	40–49	0.00	0.15	0.00	0.27	0.22	0.644
	50–59	0.00	90.74	15.64	48.41	13.95	0.522	50–59	0.00	1.55	0.67	1.27	0.41	0.708
	≥60	3.31	256.25	43.11	72.02	72.72	0.477	≥60	0.00	2.11	2.90	4.74	1.35	0.19
	**Overall**	**0.49**	**23.66**	**5.32**	**11.89**	**8.56**	0.304	**Overall**	**0.02**	**0.50**	**0.44**	**0.88**	**0.44**	**0.571**

HIV: Human immunodeficiency virus; ADCs: AIDS-Defining cancers; NADCs: Non-AIDS-Defining cancers; KS: Kaposi sarcoma; NHL: Non-Hodgkin lymphoma. * Skin includes melanoma, and liver includes hepatocellular cancer. † *p* values denote the trend analysis over the years.

**Table 3:** Standardized incidence ratios of common cancers among the PLHIV from 1990 to 2021 in Botswana.

Cancer Type	SIR (95% CI)									
	Pre-ART		Post-ART							
	1990–2001		2002–2007		2008–2012		2013–2015		2016–2021	
All	27.40	(25.77–29.10)	23.64	(22.87–24.43)	14.91	(14.42–15.40)	9.64	(9.22–10.07)	12.93	(12.51–13.36)
ADCs	30.36	(28.07–32.77)	66.29	(63.61–69.93)	34.39	(32.88–35.95)	20.76	(19.46–22.12)	36.84	(35.06–38.68)
Cervical	25.07	(21.66–28.87)	13.72	(12.41–15.12)	13.51	(12.49–14.59)	13.22	(12.07–14.45)	28.57	(26.85–30.38)
Kaposi Sarcoma	104.50	(95.00–114.70)	1449.82	(1382.39–1519.69)	388.01	(365.32–411.74)	97.54	(87.83–108.04)	144.29	(130.97–158.60)
NHL	7.49	(4.44–11.84)	39.23	(33.40–45.78)	37.35	(32.45–42.79)	18.13	(14.69–22.14)	31.88	(27.13–37.22)
NADCs	8.36	(7.56–9.21)	10.76	(10.17–11.38)	8.82	(8.40–9.27)	6.58	(6.20–6.98)	8.40	(8.03–8.78)
Breast	7.88	(6.44–9.55)	5.95	(5.08–6.91)	4.63	(4.04–5.27)	4.69	(4.03–5.42)	6.84	(6.20–7.53)
Head and Neck	20.88	(14.28–29.48)	6.87	(5.62–8.31)	8.14	(6.93–9.49)	4.40	(3.58–5.36)	9.57	(8.20–11.11)
Skin *	13.11	(9.13–18.23)	14.88	(12.20–17.96)	12.80	(10.86–14.98)	11.94	(9.93–14.24)	18.24	(15.99–20.71)
Esophagus	9.30	(5.31–15.10)	13.91	(10.59–17.95)	7.42	(6.01–9.05)	8.66	(6.99–10.59)	14.58	(12.05–17.49)
Prostate	1.51	(0.78–2.65)	6.38	(3.78–10.08)	2.41	(1.58–3.53)	3.41	(2.19–5.08)	3.88	(3.03–4.91)
Conjunctiva	NA	NA	97.15	(84.27–111.43)	86.74	(74.66–100.22)	243.79	(190.74–307.02)	540	(412.05–695.11)
Lung	7.01	(3.73–11.99)	12.06	(8.54–16.56)	4.44	(3.32–5.82)	8.16	(5.58–11.52)	15.49	(11.73–20.07)
Liver *	11.16	(6.24–18.40)	19.71	(15.30–24.98)	26.98	(21.42–33.53)	10.67	(7.78–14.27)	6.70	(4.48–9.62)

SIR: standardized incidence ratio; ART: antiretroviral therapy; NHL: non-Hodgkin lymphoma; ADCs: AIDS-defining cancers; NADCs: non-AIDS-defining cancers. * Skin includes melanoma, and liver includes hepatocellular. NA: SIR not estimable because there were zero observed cases in the HIV-uninfected reference group for that cancer–period stratum.

**Table 4:** Standardized incidence ratios of common cancers among PLHIV by sex from 1990 to 2021 in Botswana.

SIR (95% CI)											
Cancer Type	Pre-ART	Post-ART				Cancer Type	Pre-ART	Post-ART			
	1990–2001	2002–2007	2008–2012	2013–2015	2016–2021		1990–2001	2002–2007	2008–2012	2013–2015	2016–2021
	Males					Females					
ALL	87.5 (79.6–95.9)	37.2 (35.4–39.0)	22.7 (21.6–23.9)	12.2 (11.4–13.1)	14.7 (13.9–15.5)	ALL	18.2 (17.3–19.0)	14.1 (13.5–14.7)	9.8 (9.3–10.3)	12.0 (11.6–12.6)	38.3 (35.3–41.5)
ADCs	344.4 (305.1–387.4)	475.0 (447.1–504.1)	224.3 (208.5–241.0)	75.6 (67.0–85.0)	126.5 (113.5–140.5)	ADCs	32.6 (30.8–34.5)	24.2 (22.9–25.6)	17.5 (16.2–18.8)	28.1 (26.5–29.7)	56.4 (50.9–62.4)
KS	553.4 (489.1–623.7)	2630.9 (2470.2–2799.3)	1213.8 (1121.1–1312.1)	303.6 (265.1–346.0)	461.1 (406.0–521.6)	KS	886.7 (823.8–953.0)	441.4 (401.8–483.8)	105.2 (88.7–123.8)	60.2 (51.7–69.8)	354.4 (303.9–411.0)
NHL	31.0 (14.9–57.0)	43.8 (34.9–54.3)	38.8 (31.8–46.8)	18.6 (14.0–24.3)	42.8 (34.6–52.4)	NHL	46.9 (37.1–58.6)	64.4 (52.4–78.3)	37.4 (26.9–50.8)	50.5 (39.1–64.3)	32.4 (14.0–63.9)
NADCs	39.9 (34.2–46.3)	13.8 (12.7–14.9)	12.0 (11.1–12.9)	8.3 (7.6–9.1)	10.8 (10.0–11.5)	NADCs	9.9 (9.2–10.7)	8.9 (8.3–9.5)	6.6 (6.0–7.1)	7.3 (6.9–7.7)	25.1 (21.9–28.5)
Breast	*NA*	52.7 (25.3–97.0)	6.7 (2.7–13.8)	6.8 (2.2–15.8)	17.3 (10.4–27.0)	Breast	6.6 (5.6–7.7)	5.9 (5.1–6.7)	5.5 (4.7–6.4)	7.0 (6.3–7.7)	25.9 (21.0–31.7)
Head and Neck	71.2 (45.2–106.9)	7.5 (6.0–9.4)	12.1 (10.1–14.5)	5.2 (4.1–6.5)	12.9 (10.8–15.3)	Head and Neck	10.4 (6.8–15.3)	8.4 (5.9–11.6)	5.2 (3.2–8.1)	4.7 (3.3–6.5)	NA
Skin *	21.7 (8.7–44.7)	15.8 (11.1–21.9)	15.9 (12.2–20.5)	12.1 (8.6–16.4)	18.5 (14.4–23.3)	Skin *	8.8 (6.1–12.3)	6.5 (4.8–8.8)	8.9 (6.5–12.0)	14.6 (11.8–17.8)	NA
Esophagus	24.8 (12.8–43.3)	9.9 (7.3–13.2)	8.4 (6.4–10.7)	9.7 (7.5–12.3)	14.4 (11.6–17.8)	Esophagus	10.8 (5.6–18.9)	5.1 (3.5–7.2)	3.3 (2.2–4.8)	5.6 (3.7–8.0)	NA
Prostate	7.4 (3.8–13.0)	3.65 (2.16–5.77)	2.3 (1.5–3.4)	2.6 (1.7–3.9)	2.8 (2.2–3.5)	Cervical	11.5 (10.4–12.7)	13.8 (12.8–14.9)	13.5 (12.3–14.8)	25.0 (23.5–26.6)	32.6 (28.1–37.5)
Conjunctiva	NA	90.7 (72.5–112.0)	95.3 (75.9–118.2)	NA	NA	Conjunctiva	213.0 (176.3–255.0)	97.6 (79.5–118.5)	157.1 (113.7–211.5)	91.9 (62.0–131.2)	NA
Lung	49.6 (21.3–97.6)	13.2 (8.9–18.8)	7.8 (5.7–10.3)	7.1 (4.4–10.9)	13.9 (9.8–19.0)	Lung	4.1 (1.8–8.1)	1.8 (0.7–3.9)	8.0 (4.0–14.4)	3.6 (2.2–5.7)	10.1 (3.3–23.7)
Liver *	NA	2.6 (0.3–9.5)	51.7 (23.6–98.1)	19.9 (9.9–35.6)	4.1 (0.9–12.0)	Liver *	3.6 (1.2–8.4)	9.7 (4.6–17.8)	2.4 (0.7–6.2)	6.1 (2.3–13.3)	NA

SIR: standardized incidence ratio; ART: antiretroviral therapy; KS: Kaposi sarcoma. NHL: non-Hodgkin lymphoma. ADCs: AIDS-defining cancers; NADCs: non-AIDS-defining cancers. * Skin includes melanoma, and liver includes hepatocellular. NA: SIR not estimable because there were zero observed cases in the HIV-uninfected reference group for that cancer–period stratum.

**Table 5:** Cox regression analysis of risk factors associated with ADCs and NADCs.

Factor	AIDS Defining Cancers				Non-AIDS Defining Cancers			
	Crude HR (95% CI)	*p* Value	Adjusted HR (95% CI)	*p* Value	Crude HR (95% CI)	*p* Value	Adjusted HR (95% CI)	*p* Value
**Age (years)**								
18–29	*Reference*	*-*	*Reference*	-	*Reference*	-	*Reference*	-
30–39	1.58 (1.47–1.69)	<0.001	-	-	1.29 (1.19–1.39)	<0.001	1.53 (1.39–1.69)	<0.001
40–49	1.58 (1.47–1.69)	<0.001	-	-	2.05 (1.91–2.20)	<0.001	2.67 (2.43–2.94)	<0.001
50–59	0.99 (0.92–1.07)	0.834	-	-	2.78 (2.59–2.98)	<0.001	3.86 (3.51–4.25)	<0.001
≥60	0.57 (0.53–0.61)	<0.001	-	-	3.22 (3.02–3.44)	<0.001	5.23 (4.73–5.78)	<0.001
**Sex**								
Male	*Reference*	*-*	*Reference*	-	*Reference*	*-*	*Reference*	-
Female	1.73 (1.66–1.81)	<0.001	1.35 (1.27–1.42)	**<0.001**	0.80 (0.78–0.82)	<0.001	0.89 (0.86–0.93)	<0.001
**Marital status**								
Married	*Reference*	-	*Reference*	-	*Reference*	-	*Reference*	-
Single	1.82 (1.73–1.92)	<0.001	1.11 (1.03–1.19)	**0.004**	0.77 (0.74–0.79)	<0.001	1.16 (1.11–1.22)	<0.001
Prev. married	0.79 (0.61–1.01)	0.065	0.77 (0.56–1.06)	0.105	0.91 (0.79–1.04)	0.154	0.92 (0.77–1.11)	0.399
**Employed**								
Yes	*Reference*	-	*Reference*	-	*Reference*	-	*Reference*	-
No	1.12 (1.06–1.18)	<0.001	1.02 (0.96–1.09)	0.501	0.98 (0.94–1.03)	0.423	-	-
**Living with HIV**								
No	*Reference*	-	*Reference*	-	*Reference*	-	*Reference*	-
Yes	1.89 (1.74–2.04)	<0.001	1.73 (1.58–1.90)	**<0.001**	0.43 (0.41–0.45)	<0.001	0.71 (0.67–0.75)	<0.001

HIV: human immunodeficiency virus; ADCs: AIDS-defining cancers. NADCs: non-AIDS-defining cancers; CI: confidence interval; HR: hazard ratio.

## Data Availability

Restrictions apply to the availability of these data. Data were obtained from the Ministry of Health and are available from the authors with the permission of the Ministry.

## Supplementary Materials

The following supporting information can be downloaded at: https://www.mdpi.com/article/10.3390/cancers17142374/s1, Table S1: Sensitivity Analysis of HIV Prevalence Assumptions on SIR Estimates.
