# Supplementary material for: Trends in Cancer Incidence and Associated Risk Factors in People Living with and Without HIV in Botswana: A Population-Based Cancer Registry Data Analysis from 1990 to 2021
Source: Cancers (Basel). 2025 Jul 17;17(14):2374. doi: 10.3390/cancers17142374 (PMC12293140; doi:10.3390/cancers17142374)
Supplement: Supplementary file 1 [file cancers-17-02374-s001.zip › cancers-3680708_Table S1.pdf]

Table S1. Sensitivity Analysis of HIV Prevalence Assumptions on SIR Estimates.

Cancer Type	Pre-ART<br>1990-2001	Post-ART<br>2002-2007	2008-2012	2013-2015	2016-2021
All Cancers (Original)	27.40(25.77-29.10)	23.64(22.87-24.43)	14.91(14.42-15.40)	9.64(9.22-10.07)	12.93(12.51-13.36)
All Cancers (-5%)	29.56(27.81-31.40)	25.35(24.52-26.20)	15.98(15.46-16.51)	10.37(9.93-10.83)	13.93(13.48-14.40)
All Cancers (+5%)	23.92(20.15-26.86)	12.83(11.60-14.14)	12.61(11.67-13.62)	12.28(11.21-13.43)	26.52(24.92-28.20)
All Cancers (-10%)	31.97(30.07-33.96)	27.24(26.35-28.16)	17.16(16.60-17.74)	11.19(10.71-11.68)	15.04(14.55-15.54)
All Cancers (+10%)	23.64(22.24-25.11)	20.67(20.00-21.37)	13.05(12.62-13.48)	8.36(8.00-8.73)	11.19(10.82-11.56)
ADC (Original)	30.36(28.07-32.77)	66.29(64.40-69.93)	34.39(32.88-35.95)	20.76(19.46-22.12)	36.84(35.06-38.68)
ADC (-5%)	32.74(30.28-35.34)	71.03(68.16-74.00)	36.91(35.29-38.58)	22.39(20.99-23.86)	39.75(37.83-41.74)
ADC (+5%)	28.19(26.07-30.44)	61.98(59.47-64.57)	31.10(30.70-33.56)	19.27(18.06-20.53)	34.19(32.54-35.90)
ADC (-10%)	35.38(32.72-38.19)	76.29(73.21-79.48)	39.70(37.95-41.50)	24.20(22.68-25.79)	42.97(40.90-45.13)
ADC (+10%)	26.22(24.25-28.31)	58.05(55.70-60.47)	30.02(28.70-31.38)	17.91(16.79-19.09)	31.77(30.23-33.36)
NADC (Original)	8.36(7.56-9.21)	10.76(10.42-11.66)	8.82(8.40-9.27)	6.58(6.20-6.98)	8.40(8.03-8.78)
NADC (-5%)	9.02(8.16-9.95)	11.54(10.90-12.20)	9.46(9.00-9.93)	7.08(6.67-7.51)	9.04(8.65-9.46)
NADC (+5%)	7.75(7.01-8.55)	10.05(9.50-10.63)	8.25(7.85-8.67)	6.13(5.77-6.50)	7.81(7.46-8.16)
NADC (-10%)	9.76(8.83-10.76)	12.40(11.72-13.12)	10.15(9.66-10.66)	7.63(7.18-8.10)	9.76(9.33-10.20)
NADC (+10%)	7.20(6.52-7.94)	9.40(8.89-9.94)	7.73(7.36-8.12)	5.72(5.38-6.06)	7.27(6.95-7.60)
